# The effect of naproxen patches on relieving orthodontic pain by evaluation of VAS and IL-1β inflammatory factor: a split-mouth study

**DOI:** 10.1590/2177-6709.24.6.27.e1-7.onl

**Published:** 2019

**Authors:** Ladan Eslamian, Maryam Torshabi, Saeed Reza Motamedian, Yasamin Babaee Hemmati, Seyed Alireza Mortazavi

**Affiliations:** 1 Shahid Beheshti University of Medical Sciences, Dentofacial Deformities Research Center, Department of Orthodontics, School of Dentistry (Tehran, Iran).; 2 Shahid Beheshti University of Medical Sciences, Dental Biomaterials Department, School of Dentistry (Tehran, Iran).; 3 Guilan University of Medical Sciences, Dental Sciences Research Center, Department of Orthodontics, School of Dentistry (Rasht, Iran).; 4 Shahid Beheshti University of Medical Sciences, School of Pharmacy (Tehran, Iran).

**Keywords:** Pain management, Non-steroidal anti-inflammatory agents, Visual analog scale, Interleukin-1 beta

## Abstract

**Introduction:**

Pain related to orthodontic tooth movement is common and cause dissatisfaction and discomfort. **Objective:** The present study aimed to compare the efficacy of naproxen patches in pain control during orthodontic tooth separation, by means of visual analogue scale (VAS) and interleukin 1β (IL-1β) levels in gingival crevicular fluid (GCF).

**Methods:**

In this split-mouth triple-blind clinical trial, with 40 patients following separation, 5% naproxen or placebo patches were randomly placed on the upper right or left first molars every 8 hours. Pain intensity scores were determined after 2 and 6 hours, sleep time, 24 hours, days 2, 3 and 7 by the patients using a 100-mm VAS ruler. IL-1β levels in GCF were evaluated by ELISA at baseline, 1 and 24 hours and 7 days. Paired samples *t*-tests and two-way repeated measures ANOVA analysis of variance with a significance level of 0.05 were applied.

**Results:**

A total number of 30 patients (13 males and 17 females) finished the trial. Significant differences were found in pain scores (*p*< 0.0001) and IL-1β levels (*p*= 0.047) between naproxen and placebo groups. Lower pain scores were reported for the patients using naproxen patches at all time points, except 1 hour after separation. IL-1β levels were lower for the patients using naproxen patches only 1 hour after separation (*p*= 0.047). The peak of pain scores and IL-1β levels were calculated at 24 hours.

**Conclusion:**

In the light of VAS scores and IL-1β levels, naproxen patches reduced the pain caused by separator placement.

## RESUMO

**Introdução:**

a dor relacionada à movimentação dentária ortodôntica é comum e causa insatisfação e desconforto.

**Objetivo:**

o presente estudo teve como objetivo avaliar a eficácia de curativos de naproxeno no controle da dor durante a separação ortodôntica dos dentes, por meio de escalas visuais analógicas (EVA) e dos níveis de interleucina 1β (IL-1β) no fluido crevicular gengival (FCG).

**Métodos:**

neste ensaio clínico, triplo-cego, boca dividida, com 40 pacientes após a separação dos dentes, foram aplicados, de forma aleatória, curativos com naproxeno a 5% ou placebo, nos primeiros molares superiores, direito ou esquerdo, a cada 8 horas. Os escores de intensidade da dor foram registrados pelos pacientes após 2 e 6 horas, durante o sono, após 24 horas, 2, 3 e 7 dias, usando uma EVA de 100 mm. Os níveis de IL-1β no FCG foram avaliados pelo ELISA no momento inicial, e após 1 e 24 horas e 7 dias. Foram aplicados testes *t* para amostras pareadas e ANOVA de duas vias para medidas repetidas, com nível de significância de 0,05.

**Resultados:**

no total, 30 pacientes (13 homens e 17 mulheres) terminaram o ensaio clínico. Diferenças significativas foram encontradas nos escores de dor (*p*< 0,0001) e níveis de IL-1β (*p*= 0,047) entre os grupos naproxeno e placebo. Índices mais baixos de dor foram relatados pelos pacientes que usaram curativos de naproxeno em todos os tempos avaliados, com exceção de 1 hora após a separação. Os níveis de IL-1β foram menores nos pacientes que usaram os curativos de naproxeno apenas 1 ‘hora após a separação (*p*= 0,047). Os picos dos escores de dor e dos níveis de IL-1β foram registrados 24 horas após a separação.

**Conclusão:**

considerando-se os escores das EVAs e os níveis de IL-1β, pode-se concluir que os curativos de naproxeno reduziram a dor causada pela instalação dos separadores ortodônticos.

## INTRODUCTION

One of the main concerns regarding orthodontic treatment is the pain associated to the tooth movement.^1^ This pain could be discouraging and considered as one of the reasons for not participating in orthodontic treatment process. About 77% to 95% of the patients in the orthodontic clinics have reported some degree of pain and discomfort.^2^ ^,^ ^3^ Orthodontic treatment-related pain could be initiated after four hours, reaches its peak after 12-72 hours and decrease to its baseline values after 7 days.^4^ ^-^ ^6^

Some mechanisms have been proposed to explain the pain related to orthodontic tooth movement (OTM). Ischemic regions in the periodontal ligament (PDL) and mild transient pulpal inflammation are among these mechanisms.^7^ During OTM, some cytokines such as prostaglandins, substance P, histamine and leukotrienes are released and could cause pain.^8^

Interleukin 1β (IL-1β) is an inflammatory mediator that can be released as a response to the substance P^7^ and is involved in bone remodeling process.^9^ IL-1β is produced in PDL during OTM, and can be found in gingival crevicular fluid (GCF).^9^ ^,^ ^10^ It has been demonstrated that rapid OTM using elastic separators could increase prostaglandin E2 level relative to severity of initial pain and increase IL-1β levels relative to severity of delayed pain.^11^

Prostaglandins, which are inflammatory mediators necessary for OTM, are among probable pain stimulators^5^. Therefore, inhibitors of prostaglandins such as non-steroidal anti-inflammatory drugs (NSAIDs)can be prescribed to alleviate pain of orthodontic treatment.^12^ Naproxen, which is available with Diocodal and Anaprox commercial names, is one of the NSAIDs that can prohibit production of prostaglandins and leukotrienes.^13^ Therefore, its efficacy in orthodontic pain management has been evaluated in some studies.^14^ ^-^ ^17^ Although it has been reported that naproxen is an effective painkiller during OTM, its efficacy during local delivery as mucosal patches has not been studied.

Several medications and other approaches such as low level laser therapy, chewing gum, local prescription of gels and patches, vibratory and transcutaneous electric nerve stimulation^6^ ^,^ ^18^ ^-^ ^22^ have been introduced for pain control during orthodontic treatment; however, no general consensus has been reached. The most appropriate method should be efficient with minor or no harm for the patient. Therefore, the current study was performed to compare the efficacy of naproxen patches in pain control during orthodontic tooth separation, using the visual analogue scales (VAS) and IL-1β levels in GCF. Naproxen patches were used for local delivery of the drugs, in order to increase its efficiency as well as reducing the dosage of the drug and possible risks of systemic prescription. 

## MATERIAL AND METHODS

### Study design

This triple blind split-mouth randomized controlled clinical trial was performed on patients referred to the orthodontic department (Shahid Beheshti University of Medical Sciences) for fixed orthodontic treatment in 2016. Study protocol was according to CONSORT statement 2010, and was approved by the ethical committee of the university. Also, informed consent was taken from all included patients.

### Patients

Adult patients (age ≥ 18 years) who needed banding of upper first molars for fixed orthodontic treatment were enrolled consecutively from study population. The inclusion criteria were: generally healthy patients, no periodontal or endodontic disease, presence of bilateral first and second maxillary molars and second maxillary premolars, tight posterior teeth contacts with no spacing, presence of antagonist teeth without posterior open bite, and no psychologic or mental disorder. Patients who did not participate in the follow up sessions, had no pain on both sides, took systemic or topical painkillers except prescribed patches, removed separators, did not used patches as prescribed, and did not fill VAS questionnaire on time or lacked cooperation were excluded. The sample size of 23 was calculated to find 1 unit difference with 1.1 unit standard deviation between two groups and considering α=0.05, β=80%, Z_α_=1.96 and Z_β_=0.84. Sample size was increased to 40 patients in order to increase study power and considering probable dropouts.

### Preparation of naproxen patch

Naproxen patches were prepared using solvent casting method: 2.5% HPMC and 1% PVL were used for adhesion to the oral mucosa; also, 4% propylene glycol was used for ligation of naproxen, and 0.25% aspartame was used to sweeten the patches. Ingredients plus naproxen (South China pharmaceutical company, Shenzhen, China) were solved in chloromethane and alcohol solvents with 2:1 ratio and kept under fume hood for 72 hours. In order to evaporate the remaining solvents, the fabricated patches were dried for one more week in the room.

The proper size of the patches was determined following try and error method: 0.5 x 1.5-cm patches seemed to be the most appropriate size for adhesion and remaining in place. 

### Tooth separation

Two sets of patches were prepared and coded: one set contained 5% naproxen, and the other set without naproxen (placebo). The patches and their packages were visually similar and only a code was written on each package. So, neither the examiner nor the patient were aware of the type of the patches (double blind). Maxillary first molars were separated from adjacent teeth using elastic separators (American Orthodontics, Monrovia, CA, USA). One package of each set was given to the patient. Then, the patient was instructed to place one patch from one of the packages on the attached and free gingiva of maxillary first molar of one side and one patch from the other package on the other side molar. The sides were randomly selected using coin flip method. Patient’s age, gender as well as code of the patch used on each side were recorded. The patients were instructed to place the patch on the gingiva, press it and hold it for 20 seconds every 8 hours for 7 days. There was no limitation for eating and drinking. Also, the patients were instructed to use painkillers if they had intolerable pain.

### Outcome measurement: pain

The patients were instructed to record the pain they felt on each side after 1 and 6 hours, at night, 24, 48 and 72 hours and 7 days, based on a 100-mm VAS ruler. A questionnaire including 21 VAS rulers and instruction were given to the patients. Zero indicated no pain, while 100 showed the most severe pain.

### Outcome measurement: IL-1β

**Figure 1:**
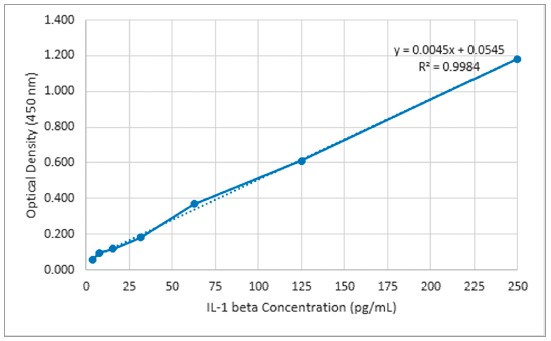
Standard curve of human IL-1β.

Levels of IL-1β in GCF was measured at the time of tooth separation (baseline) and after 1 and 24 hours and 7 days, using ELISA. First, GCF was collected from distobuccal and mesiobuccal sulcus of maxillary first molars using paper cones (ARIA DENT Tehran, Iran) size 30 after isolation with cotton roll. For this, paper cones were inserted 1 mm into gingival sulcus for 60 seconds. Then, paper cones were transferred to sterile 1.5-ml µtubes containing 200 µl phosphate buffered saline for couple of minutes followed by 10-minute centrifuge at 3000 rpm at 4°C. Finally, the supernatant fluid containing GCF proteins was transferred to another sterile µtube, which was frozen at -70°C. Commercial ELISA kit (GMBH, Berlin, Germany) was used to measure the levels of IL-1β in the GCF samples, following manufacturer’s instructions. Briefly, the kit micro-wells including human anti-IL were rinsed twice with 400 µl PBS. Then, 50 µl buffer and 50 µl of each sample plus 50 µl Biotin-conjugated anti-human IL added to each well and incubated for 2 hours at room temperature. The wells were rinsed 3 times and received 100 µl Streptavidin-HRP enzyme and incubated one more hour. Again, the wells were rinsed and 100 µl substrate was added to each well and incubated for 10 minutes. Finally, 100 µl stop solution was added to each well and color to the wells containing IL, which turned yellow. Optical density was read using ELISA reader (Anthos2020, Berlin, Germany) at 450 wavelengths. Finally, density of IL-1β (pg/ml) was determined using standard absorbance values (Fig. 1).

### Statistical analysis

The results of pilot study were compared using repeated measure ANOVA. A two-way repeated measure ANOVA was used to analyze the effect of study group, time, as well as pain scores or levels of IL-1β. To compare patients pain scores or IL-1β levels at each time point between the two groups, paired samples *t*-test was applied. To evaluate association of pain scores and levels of IL-1β, Pearson correlation coefficient and regression model was used.

All statistical analyses were performed using SPSS computer software (SPSS Inc., Chicago, IL, USA), with a significance level of 0.05, by an expert who was blind to the allocated groups (triple blind).

## RESULTS

A total of 40 patients (23 females and 17 males) were enrolled in the study, out of which 10 patients were excluded. The reasons for exclusion were: no regular use of the patches (2 patients), lack of pain at both sides (7 patients) and use of painkiller during the experiment (1 patient). Therefore, a final number of 30 patients (13 males and 17 females) with a mean age of 27.47±4.08 years (22 to 40 years) finished the study.

### Pain

The mean and standard deviation (±SD) of pain scores based on VAS at different time points in both groups are shown in Table 1. Two-way repeated measure ANOVA revealed that groups (naproxen vs. placebo) (*p*< 0.0001), time (*p*< 0.0001) and their interaction (*p*= 0.005) had a significant effect on the pain scores. Comparison of the mean pain scores at each time point between two groups revealed significantly lower pain score at naproxen group at each time point (*p*< 0.05) except at 1 hour after separation (*p*= 0.792).

**Table 1:** Comparison of the mean pain scores at different time point between naproxen and placebo patch groups (sample size: 30).

Group		Time point							
		1h	6h	At night	24h	2nd day at 6pm	48h	72h	7 days
Placebo	Mean	17.67	27.83	32.17	36.50	32.00	26.83	21.67	14.50
	SD	14.840	20.286	21.483	23.087	20.536	18.959	18.770	14.701
Naproxen	Mean	18.17	21.33	26.00	27.33	23.50	18.17	15.67	10.33
	SD	16.891	17.267	18.213	20.331	18.059	14.650	14.126	9.820
P value		0.792	< 0.001*	< 0.001*	< 0.001*	0.001*	0.001*	0.016*	0.026*

* Significant at 0.05 using paired samples t-test.

There was no difference in the mean pain scores between males and females (*p*= 0.29).

### Level of IL-1β

The mean and standard deviation of levels of IL-1β at different time points in both groups are demonstrated in Table 2. Two-way repeated measure ANOVA revealed that groups (naproxen vs. placebo) (*p*= 0.047), time (*p*< 0.0001) had a significant effect on the levels of IL-1β. However, their interaction was not statistically significant (*p*= 0.3). Comparison of mean levels of IL-1β at each time point between two groups revealed that only 1 hour after separation naproxen group had significantly lower levels of IL-1β, compared to placebo group; while the difference at other time points was not significant (*p*> 0.05).

**Table 2:** Comparison of the mean IL-1β levels at different time points between naproxen and placebo patch groups (sample size: 30).

Group		Time point			
		baseline	1h	24h	7 days
Placebo	Mean	68.69	87.83	102.66	74.18
	SD	4.33	5.34	7.17	5.48
Naproxen	Mean	72.37	74.22	93.20	64.67
	SD	5.37	5.18	5.21	4.64
P value		0.552	0.047*	0.253	0.115

* Significant at 0.05 using paired samples t-test.

There was no difference in the mean IL-1β levels between males and females (*p*= 0.92).

### Association of pain and level of IL-1β

Pearson correlation coefficient showed significant positive correlation between pain scores and IL-1β levels in control group after 1 hour (*p*= 0.04 and r = 0.374) and a negative correlation between them in naproxen group after 7 days (*p*= 0.03 and r = -0.395) (Table 3).

**Table 3:** Association of pain scores and IL-1β levels in naproxen and placebo patch groups (sample size: 30).

Group		Time point		
		1h	24h	7 days
Placebo	Pearson Correlation	0.374	0.04	0.015
	P value	0.04*	0.83	0.94
Naproxen	Pearson Correlation	0.077	0.144	0.395
	P value	0.69	0.45	0.03*

* Significant at 0.05.

Linear regression model revealed that each 10 unit increase in IL-1β levels is related to 2 unit increase in the pain scores. Therefore, the following formula could be used to show correlation between pain scores and IL-1β levels: Pain (VAS) = 1 ± 0.2 IL-1β (pg/ml)

## DISCUSSION

NSAIDs are appropriate choices for pain management during orthodontic treatment, as the pain is related to combination of pressure, ischemia and inflammation.^23^ ^,^ ^24^ The current study was performed to evaluate the effect of naproxen patch on separator placement pain relief. The results revealed that patients felt less severe pain on the side that received naproxen patch, compared to the placebo side, at every time point, except 1 hour after tooth separation.

NSAIDs could have various complications^25^ ^,^ ^26^ such as gastrointestinal problems.^27^ They inhibit cyclooxygenase enzyme and reduce production of prostaglandins, which have a preservative role in gastrointestinal mucosa. Therefore, their administration should be limited. The current study evaluated the effectiveness of local delivery of naproxen in order to reduce systemic complications. Locally administered naproxen was used in a recent study by Eslamian et al.^14^ The naproxen gel which they used have several limitations such as rapid wash out by saliva and various dosage while applying the gel on the desired site. The naproxen patches used in the current study could release the drug up to 2 hours after application and had exactly the same dosage of naproxen, as they all had the same size. Although variety in gingival thickness could cause different levels of drug absorption released from patches, the split-mouth design was applied to reduce the effect of this possible confounding factor.

Comparison of mean pain scores between the two groups showed significantly lower pain in the naproxen group at every time point, except 1 hour after separation. These results are in agreement with some previous studies.^14^ ^-^ ^17^ ^,^ ^28^ Eslamian et al.^14^ evaluated the effectiveness of 5% naproxen gel administered every 8 hours for 3 days on pain management related to elastic separators in a split-mouth study. They concluded that naproxen gel significantly reduced pain scores assessed by VAS at every time point from 1 hour to 7 days. Two studies by Polat et al.^16^ ^,^ ^17^ showed that single dose naproxen was able to decrease the pain of fixed archwire activation and it was more effective that other analgesics during the first day. However, Patel et al.^15^ reported that administration of naproxen 1 hour before and 3 and 7 hours after separation did not significantly reduce patients’ pain. A recent systematic review reported that naproxen has a significant effect in OTM pain relieve at 6, 12, and 24 hours.^28^ The cited review also concluded that the timing of administration could significantly influence the effectiveness of naproxen.

The results of the current study showed that the mean pain score in both groups reaches its peak after 24 hours and then decrease until 7 days. It seems that the most prominent inflammatory responses during OTM happen during the first day.^29^ ^,^ ^30^ Previous studies also reported highest pain after 24 hours.^17^ ^,^ ^23^ ^,^ ^31^ ^-^ ^33^ Also, similar to the previous studies,^14^ ^,^ ^15^ ^,^ ^19^ ^,^ ^34^ ^,^ ^35^ there was no significant difference between pain experienced by males and females in this study.

The pain model and assessment of pain using VAS are common methods in orthodontic literature. Placement of elastic separators move the teeth and can cause various degrees of pain. Compared to archwire activation, placement of separators allows more control on the confounding factors.^19^ The VAS also have been demonstrated to have an acceptable precision and validity for assessment of subjective pain^36^ ^-^ ^38^ and could be used for comparison of pain between groups.^39^

It has been revealed that IL-1 levels increase during experimental tooth movement, reaching its peak after 1 day and recovering to the normal value during 1 week.^40^ Also, previous human studies showed up-regulation of IL-1 after 24 hours during OTM.^10^ ^,^ ^11^ ^,^ ^31^ ^,^ ^32^ Since the peak pain intensity and IL-1 level during OTM occurs simultaneously after 24 hours, in the current study the level of IL-1 was considered as an indicator of pain intensity. However, Gameiro et al.^32^ reported that IL-1 levels and pain scores were not statistically correlated at every time point. The results showed that, similar to the previous studies,^31^ ^,^ ^32^ levels of IL-1 reached its peak after 24 hours, at which the pain scores were also at their highest level. However, statically significant but weak correlation between levels of IL-1 and the pain score was found only at 1 hour after separator placement in the control group and after 7 days in the naproxen group. Lack of correlation between IL-1 level and pain score might be the reason for non-significant difference in IL-1 levels between the two groups after 1 and 7 days. Gameiro et al.^32^ stated that IL-1 could not be used as the sole measurement for assessment of pain during OTM. Future studies should assess levels of other cytokines in GCF to find the one that have stronger correlation with pain intensity.

## CONCLUSION

The pain scores in the naproxen group were lower than placebo groups at all time points from 6 hours to 7 days after separation. However, the difference was not significant after 1 hour.

Levels of IL-1β was lower in naproxen group at 1 hour after separation, but no difference was found at later time points until 7 days. Lack of strong correlation between pain scores and IL-1 levels indicates that there is a need for other biologic indicators of pain intensity during OTM.

As the highest pain intensity was recorded one day after separation and naproxen patches were effective in pain relief, it could be concluded that naproxen patches could be administered specially at the first days after orthodontic separator placement.

This study showed that locally administered naproxen patches with lessened dosage can successfully relief pain and there is no need for systemic usage of the drug. 
